# Lamb Performance on Island Pastures in Northern Norway

**DOI:** 10.3389/fvets.2020.00402

**Published:** 2020-07-14

**Authors:** Vibeke Lind, Øystein Holand, Finn-Arne Haugen, Geir Steinheim

**Affiliations:** ^1^Norwegian Institute of Bioeconomy Research (NIBIO), Ås, Norway; ^2^Department of Agriculture and Aquacultural Sciences, Norwegian University of Life Sciences, Ås, Norway

**Keywords:** daily gain, vegetation types, stocking rate, grazing quality, sheep

## Abstract

The Norwegian sheep industry is based on utilization of “free” rangeland pasture resources. Use of mountain pastures is dominating, with about two million sheep grazing these pastures during summer. Regional challenges related to e.g., loss of sheep to large carnivores make farmers think differently. The Norwegian coastline is among the longest globally and is scattered with islets and islands. Alone along the coast of Nordland county, it is estimated more than 14,000 islands. Use of islands for summer pasture is an alternative but there is a limited knowledge about such a management system. In this study, we examined lambs' average daily gain on island pastures at the coast of Norway. In total 230 lambs on three islands (Sandvær, Sjonøya, and Buøya), with varying pasture quality and stocking rate, for 3 years (2012, 2013, and 2014). At Sandvær as much as 92% of the island was characterized as high nutritional value while at Sjonøya and Buøya only 15%, was characterized high nutritional value. We found an average daily lamb growth rate of 0.320 kg d^−1^. Lambs on Sandvær had a higher daily gain (*P* < 0.05) than those on Sjonøya and Buøya, and lambs' average daily gain was significantly lower (*P* < 0.05) in 2013 compared to 2012 and 2014. We conclude that with a dynamic and adaptive management strategy there is a potential to utilize islands for sheep grazing during summer.

## Introduction

The Norwegian sheep industry is based on utilization of spatially diverse rangeland pasture resources as reflected in different management systems and local adaptations. Only 3% of Norway is used for crop production, but more than half of the land area has potential value as livestock pasture. Rekdal (1) estimated that harvesting of rangeland vegetation by livestock could be doubled and in a White paper from the Norwegian Government from 2016 (2) an increase in rangeland grazing is encouraged for all regions of the country. In Norway, ~2 million sheep are released onto extensive pastures for summer grazing (3). Most sheep are grazing rangeland pastures in mountainous areas but challenges due to high mortality to e.g., large carnivores have increased the interest in utilizing pastures on islands and islets along the coast.

The coastal line of Norway's mainland is estimated to about 30,000 km, but including islands, the length increases to about 103,000 km (4). Nordland county, stretching from 65 to 69°N, has a surface area of about 38,000 km^2^ and constitutes 12% of the total area of Norway (5). Nordland coastal line is estimated to be about 27,000 km of which 21,000 km are island coastal lines. The coast is scattered with some 18,000 islands of all sizes, from small islets of ~1 ha to inhabited islands up to 500 km^2^ (5). Many of the smaller islands were previous inhabited but are now abandoned and traditional farming with meadow harvesting and livestock grazing has ceased. Indeed, the open and grazing-induced semi-natural pastures rich in biodiversity and pleasing to the human's eye are at risk. In Nordland county, farmers are therefore offered a diverse package of subsidies and incentives for restoring and maintaining this unique semi-natural coastal landscape (6).

Most of these islands are flat (rising to 40–50 m above sea level) and natural fresh water supply can be limited during summer. The phenological development of the plants is more uniform on islands than in mountain areas. Vegetation types, their proportion, and distribution and thus pasture value varies substantially between islands (7). A management of stocking rate customized to available pasture resources is therefore necessary to ensure animals' performance and welfare (8). However, appropriate stocking rates are defined by decades-long experience by farmers, while little scientific knowledge exists about sheep performance on these coastal pastures.

In a field study we investigated lamb performance during three consecutive summer grazing seasons (2012–2014) on three islands, with highly variable grazing values and stocking rates at the coast of Helgeland, Nordland county. The aim of the study was to describe lamb daily weight gain and to evaluate and discuss opportunities and challenges for future sustainable sheep grazing on island pastures.

## Materials and Methods

### Ethics Statement

The study was performed at commercial farms and the only extra handling of animals was through weighing. The animals were collected by help of sheep dogs per normal practice at the farms. We followed the regulation for use of animals in experiments, adopted by the Norwegian Ministry of Agriculture and Food and approved by the administrative officer for animal trials of NIBIO (Approved Animal welfare unit no 171). Ethical review and approval was not required for the animal study because the study was performed at commercial farms. Written informed consent was obtained from the owners for the participation of their animals in this study.

### Study Area

**Figure 1:**
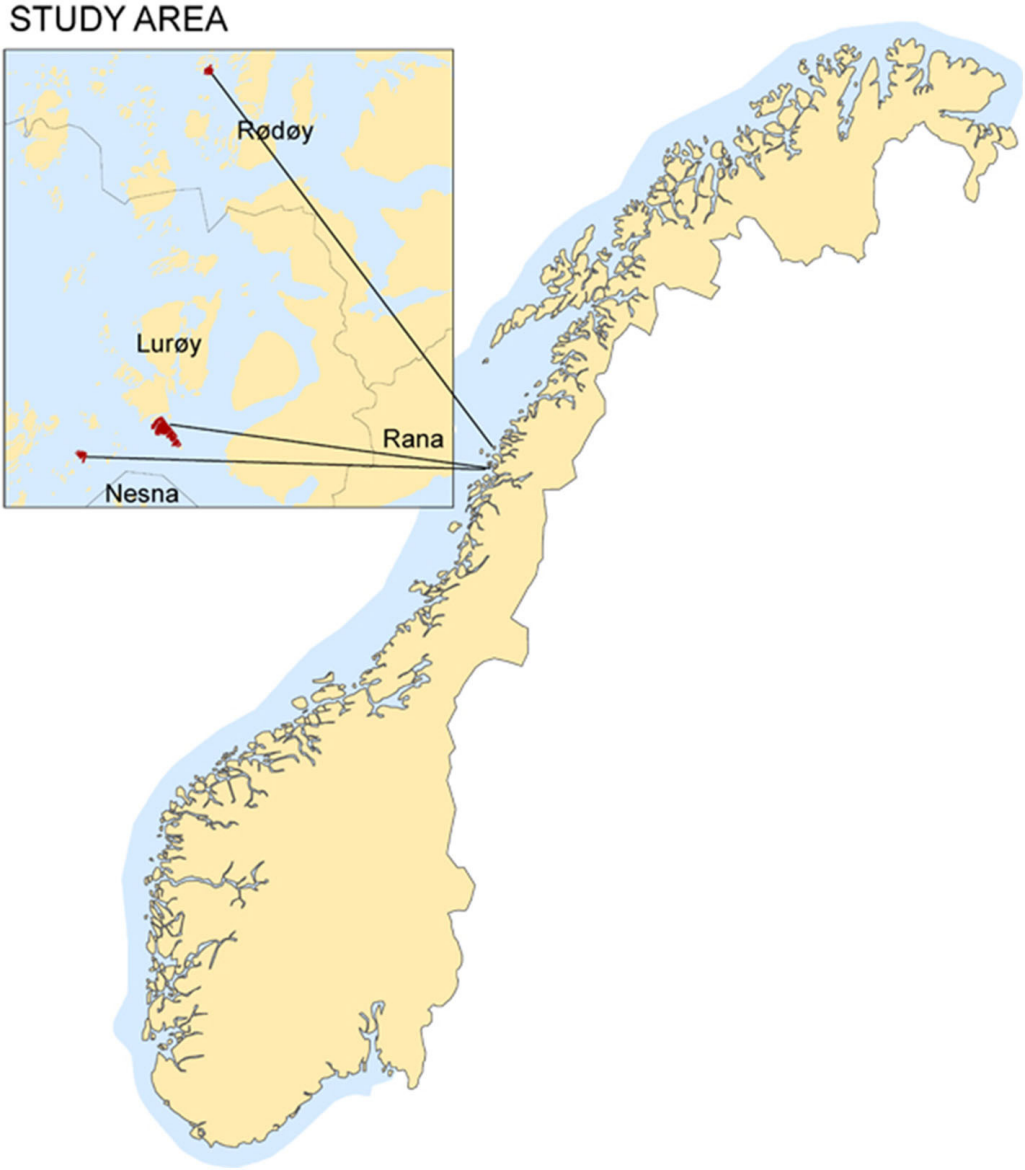
Map of Norway and the islands (insert) Sandvær (south), Sjonøya and Buøya (north).

The three islands studied have been used for sheep grazing during many years. The islands are situated in Lurøy and Rødøy municipalities, at the coast of Nordland county in Norway (Figure 1). Sandvær (66°20′35 N, 12°43′55 E) covers 39 ha and range up to 20 meters above sea level (m.a.s.l.). Sjonøya (66°21′51 N, 12°52′42 E) covers ~208 ha and range up to 40 m.a.s.l while Buøya (66°37′31 N, 12°56′35 E) covers 36 ha and range up to 40 m.a.s.l. The total livestock unit (LU) at Sandvær, Sjonøya, and Buøya were 1.26, 3.60, and 2.70, respectively, in all 3 years (2012, 2013, and 2014). At Sjonøya, an additional 40 sheep of the Old Norwegian breed (5.6 LU) grazed and was included when stocking rate was calculated. The weather is typical coastal climate with mild winters and wet summers, with mean temperature during winter around 0°C and during summer around 12°C (9). Annual precipitation is around 2,000 mm.

### Vegetation

The vegetation was mapped using the system of Rekdal and Larsson (10) and a total of 19 different vegetation types, both natural and semi-cultivated, were identified on the islands (Table 1). We classified the vegetation types into four main classes based on value for sheep grazing: “Not Suitable” (no grazing value or inaccessible), “low,” “medium,” or “high,” following the vegetation classification system of Rekdal (7). The “Not Suitable” class including barren land and exposed bedrock was omitted from all analyses. Rekdal (7) evaluate the grazing value of the different vegetation types based on plant production and the grazing habits of the livestock species. At present, there is no systematic information on nutritional values such as energy, protein, and fiber for different vegetation types (11) and such values would necessarily be highly uncertain due to varying plant species composition within a vegetation type, site-specific phenological development for each plant species, and the impact on the vegetation from the grazing animals both within year and historical.

**Table 1:** Distribution of vegetation types and nutritional value in area (ha) and percentage (%) at Sandvær, Sjonøya, and Buøya.

**Vegetation type**	**Grazing value**	**Sandvær**		**Sjonøya**		**Buøya**	
		**Ha**	**%**	**Ha**	**%**	**Ha**	**%**
Dwarf shrub heath	Medium			0.25	0		
Low herb meadow	High	14.7	38	5.1	2		
Tall forb meadow	High	8.2	21				
Lichen and heather	Low			0.1	0		
birch forest							
Bilberry birch forest	Medium			5.3	3		
Meadow birch forest	High			2.6	1		
Pasture land forest	High			1.2	1		
Meadow spruce forest	Medium			0.3	0		
Poor swamp forest	Low			0.8	0		
Rich swamp forest	Medium			0.7	0		
Bog	Low			4.1	2	3.7	10
Fen	Low	1.3	3	6.1	3	0.4	1
Sedge marsh	Low						
Coastal heath	Low	1.9	5	64.5	31	8.6	24
Damp heath	Low			85.3	41	18.2	50
Moist meadows	High	1.5	4	2.3	1		
Pasture	High	10.8	28	19.9	10	5.0	14
Barren land							
Exposed bedrock		0.3	1	9.2	4	0.4	1

In the study area, vegetation types of high nutritional value, contain species such as common bent (*Agrostis capillaris*), sweet vernal grass (*Anthoxanthum odoratum*), kentucky bluegrass (*Poa pratensis*), and red fescue (*Festuca rubra*). Wavy hair-grass (*Deschampsia flexuosa*), blueberry (*Vaccinium myrtillus*), and sweet vernal grass (*Anthoxanthum odoratum*) are found in medium nutritional value classes while the low nutritional value class is dominated by crowberry (*Empetrum nigrum*), heather (*Calluna vulgaris*), and purple moor-grass (*Molinia caerulea*).

**Figure 2:**
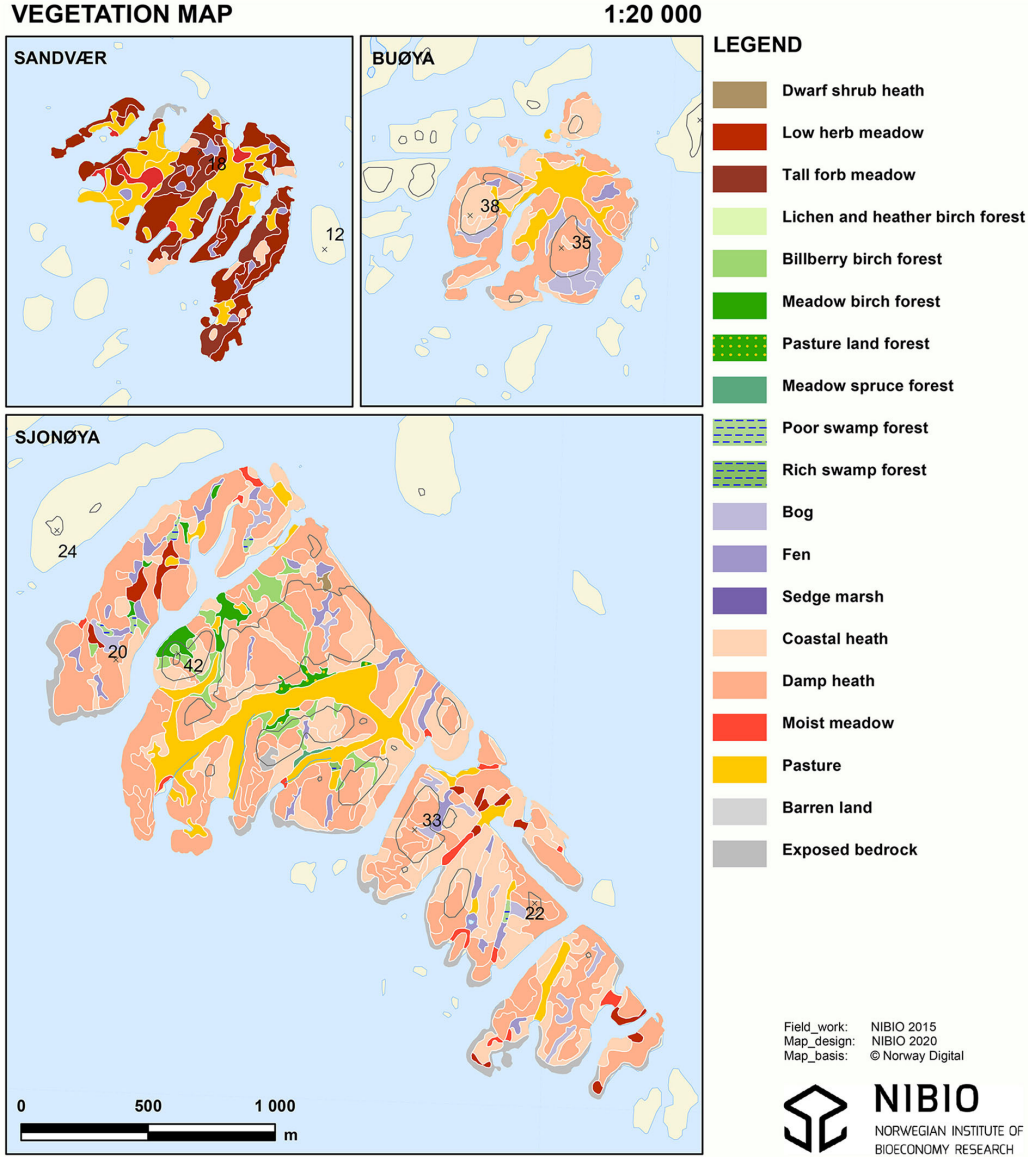
Vegetation maps of Sandvær, Sjonøya, and Buøya.

Table 1 shows the distribution and proportion of the vegetation types and their nutritional classes on the three study islands. Figure 2 shows the vegetation maps of the three islands.

### Animals

The study animals were of the dominating sheep breed in Norway, the cross-bred, prolific Norwegian White Sheep. Ewes and lambs were recruited from two commercial sheep farms that had used the islands for summer grazing during several years prior to the study. We asked the farmers to randomly select adult ewes (>2 years of age) with two lambs at foot. Twins are the most common litter size in the breed. The farmers selected the animals post-lambing to ensure that all ewes and lambs were healthy and distributed the animals randomly to the islands (Table 2). The animals had access to all vegetation types within each island, and did not receive any supplement feeding during the grazing period.

**Table 2:** Number of Norwegian White ewes and lambs at Sandvær, Sjonøya, and Buøya in 2012–2014.

	**Sandvær**		**Sjonøya**		**Buøya**	
	**Ewes**	**Lambs**	**Ewes**	**Lambs**	**Ewes**	**Lambs**
2012	7	13	20	40	15	30
2013	7	14	20	40	15	30
2014	7	14	20	40	12	23

Due to missing data the number of lambs used in the performance analysis was 11, 13 and 14 at Sandvær for 2012, 2013, and 2014, respectively. For Sjonøya, 39, 39, and 40 lambs are included in the analyses and for Buøya 28, 28, and 18 for 2012, 2013, and 2014, respectively.

Lambs were born in May and were between 1 and 4 weeks old when released to the island pastures. All ewes and lambs were individually ear-tagged for identification. The ewes were weighed before released to and when collected from the islands. The lambs (*n* = 230) were weighted at birth (average 4.90 Standard deviation, *SD* = 0.86 kg), when released to the island (average 9.98 *SD* = 3.51 kg), and when collected (average 38.1 *SD* = 7.90 kg) as normal routine done by the farmers. The animals were on average released to the islands in week 21 (end of May) and collected in week 37 (beginning of September).

### Weather Conditions

As a proxy for the daily average temperature and precipitation at the three islands, data was collected from the weather station at the mainland at NIBIO Tjøtta in Nordland County (65°49'22N, 12°25'37E); the information is shown in Table 3 for June, July, and August of the years 2012, 2013, and 2014.

**Table 3:** Average temperature (°C) and total precipitation (mm) in June, July, and August for 2012, 2013, and 2014 at Tjøtta weather station.

	**June**		**July**		**August**	
	**Average temperature**	**Total precipitation**	**Average temperature**	**Total precipitation**	**Average temperature**	**Total precipitation**
2012	10.9	15.0	12.4	102.3	12.2	47.2
2013	13.0	84.1	13.3	169.2	13.9	65.6
2014	12.0	36.8	18.8	63.8	15.1	70.5

### Statistics

Data on a total of 230 twin lambs were analyzed by fitting a general mixed linear model in Proc Mixed of SAS statistical software (12), using the Satterthwaite option for estimation of denominator degrees of freedom. The model used was

$$\begin{array}{l} \text{y}=\text{Xb}+\text{Zu}+e, \end{array}$$

where y is the observation of individual lamb body growth (kg d^−1^) on island pasture; *b* is a vector containing fixed demographic and environmental effects, and *X* is the incidence matrix relating the observations to the effects in b. The random effect of ewe by year is *u*, related to observations by incidence matrix *Z*. Finally, *e* is the residual variance.

The effects in *b* are

- the overall average daily weight gain μ.
- regression variables lamb age (days) at start of island grazing (2–58; mean 15.8), early lamb growth (kg d^−1^) from birth to start of island grazing (−0.05-0.77; mean 0.33), and ewe weight (kg) at the start of island grazing (41–101; mean 73.9),
- class variables lamb sex (female or male, sex ratio 0.5), ewe age in years (1, 2, …,6; mean 2.5), island (Sandvær, Sjonøya, or Buøya), year (2012, 2013, or 2014), and the interaction effect between year and island (nine levels).

To account for dependency within litters, the interaction effect of individual ewe by year was fitted as random, with 121 classes (98 ewes). Seventeen ewes were observed in more than 1 year; but the litters of these were still treated as independent of each other. Least square means were estimated for all significant fixed class variables and pair-wise *t*-tests were performed. Effects were considered significant when *P* < 0.05.

## Results

As much as 92% of the area of Sandvær is characterized as high nutritional value which here includes the vegetation types of low herb meadow, high forb meadow, moist meadow, and pasture. At Sjonøya, about 80% of the area is characterized as low nutritional value with the island dominated by coastal heath (31%) and damp heath (41%). Most of the remaining area is classified as medium to high nutritional value (low herb meadow, meadow birch forest, and pasture). At Buøya six vegetation types were present and the island is dominated by low nutritional value classes (86%). On this island, high nutritional value is only found on patches of pasture (14%). Exposed rock is found on all islands, 1% at both Sandvær and Buøya and 4% at Sjonøya.

Lambs' average daily gain on the island pastures was 0.320 kg d^−1^ (*SD* = 0.067 kg d^−1^), and they spend on average 89 days on the islands (*SD* = 13 days). From the mixed model (Table 4) all variables in the model were significantly affecting lamb growth at *P* < 0.05, except for lamb age (days) at release on the islands (*P* = 0.66).

**Table 4:** Effect of lamb age (d) at release, lambs average daily gain (g d^−1^) from birth to release, lamb sex (male or female), age of ewe (year), ewe weight (kg) at release, islands (Sandvær, Sjonøya, Buøya), year (2012, 2013, 2014), and the interaction between year and island, their nominator Degrees of Freedom (NDF), denominator Degrees of Freedom (DDF), *F* and *P*-values.

**Effect**	**NDF**	**DDF**	***F*-value**	***P***
Lamb age	1	101	0.19	0.663
Early growth	1	209	6.64	0.011
Sex	1	206	27.81	<0.001
Ewe age	5	104	2.69	0.025
Ewe weight	1	104	13.37	<0.001
Island	2	101	20.88	<0.001
Year	2	106	10.79	<0.001
Year*Island	4	102	32.07	<0.001

Least square means (*LS* means) for the class variables island, year, interactions of year ^*^ island, lamb sex, and ewe age are shown in Table 5.

**Table 5:** Least squared means (LS means) corrected for the other effects in the model for lambs average daily gain (kg d^−1^) on island pasture, with standard error (SE), for class variables island, year, year * island, and sex in the mixed model.

**Effect**	**Level**	**LS means**	***SE***
Island	Sandvær (Sa)	0.372	0.013
	Sjonøya (Sj)	0.285	0.007
	Buøya (Bu)	0.326	0.007
Year	2012	0.344	0.009
	2013	0.303	0.008
	2014	0.337	0.008
Year*Island	2012*Sa	0.472	0.016
	2012*Sj	0.230	0.010
	2012*Bu	0.331	0.014
	2013*Sa	0.301	0.023
	2013*Sj	0.302	0.011
	2013*Bu	0.306	0.013
	2014*Sa	0.345	0.015
	2014*Sj	0.324	0.008
	2014*Bu	0.342	0.013
Sex	Male	0.343	0.006
	Female	0.313	0.007

*T*-tests between *LS* means showed that lamb daily gain (Table 5) differed between islands (*P* < 0.01) and that lambs at Sandvær had the highest daily gain (0.372 kg d^−1^) mainly caused by the high growth rate in 2012. Across islands, lamb daily gain was higher in 2012 and 2014 compared to 2013 (*P* < 0.01). The interaction between year and island show that the lambs' growth on Sandvær in 2012 was higher than that of all other year ^*^ island classes (*P* < 0.01); no other significant differences were found. Male lambs had a higher average daily gain than female lambs (*P* < 0.01).

Daily weight gain of lamb from birth to release on the islands was fitted as a regression variable, with 0.076 (*SE* 0.029) kg d^−1^, i.e., an increase in early growth of 0.1 kg d^−1^ would give an increased growth on island of 0.0076 kg d^−1^. Given 89 days grazing period on the islands, this increase gives an extra ≈ 0.7 kg live weight per lamb. The regression on ewe weight was 0.002 kg d^−1^ (*SE* 0.0005) per kg ewe live weight; meaning that 1 kg higher ewe weight corresponds to an increased lamb growth of 2 g per day or 178 g during the 89 days grazing period on the islands. The variance component of year by ewe, of 0.0005 (*SE* 0.0002) was significantly different from zero (Wald-test: *Z* = 2.50, *P* = 0.006); the residual variance was 0.0013 (*SE* 0.0002) and different (*Z* = 7.45, *P* < 0.001) from zero.

## Discussion

In the present study, we investigated lambs' performance when grazing semi-natural pastures on islands to evaluate the quality of these pastures. Further, we corrected for age of ewe, weight of ewe, and sex of lamb. All these effects significantly influenced lambs' average daily gain, as expected (13, 14) and are therefore not considered in the following discussion.

The proportion of vegetation types of high nutritional value differed between the islands. The vegetation type pasture is mainly former managed permanent grassland for forage production, now abandoned, and has a high nutritional value with an estimated grazing capacity of 0.75 LU per ha per year (7). At Sandvær, 35.5 ha, around 92% of the total area was classified as high value according to Rekdal (7). Pasture alone, covering around 12 ha, could sustain around 3.6 LU. In addition to pasture, the high nutritional value vegetation classes low herb meadow (covering 38%) and tall forb meadow (covering 21%) were found on this island. During the 3 years experiment, only 21 sheep (1.26 LU) grazed the island every summer. When vegetation is grazed at an optimum stocking rate the forage quality maintains. However, when the number of animals is too low to maintain the quality, the non-grazed areas will degrade. At Sandvær, the tall forb meadow vegetation type was dominated by meadowsweet *(Filipendula ulmaria)* resulting in a degradation of its grazing value. Meadowsweet has little grazing value for sheep and is often seen dominating areas with zero or low grazing pressure (15). The higher lamb daily gain at Sandvær compared to the two other islands, could be attributed to the high percentage of vegetation types with high nutritional value. A higher stocking rate combined with an earlier release would help improve the now low nutritional value of the vegetation type tall forb meadows and lead to an even higher weight gain of the lambs.

The stocking rate at Sjonøya was estimated to 9.2 LU including the flock of Old Norwegian (3.6 Norwegian White and 5.6 Old Norwegian). About 10% (20 ha) of the island was pasture which could carry about 10 LU (7). In addition, the high nutritional vegetation types of low herb meadow, meadow birch forest, pasture land forest and moist meadows (in total covering 5% of the area) was present allowing additionally 2 LU to graze the island. The number of grazing sheep at Sjonøya during the summer was equivalent to 9.2 LU thus close to estimated grazing capacity of 10 LU. However, lamb daily gain on Sjonøya was significantly lower than that of both Sandvær and Buøya. Sjonøya consists of four smaller islands connected only at low tide. Most of the cultivated pasture type is located on one of them and sheep could be temporary stranded at an island with mostly low nutritional value vegetation types. This could be one explanation for the lower average daily gain. The Old Norwegian sheep present at Sjonøya is a breed which can utilize coastal heath when higher nutritional value of other forages become scarce. However, during summer when higher nutritional value is available, the Old Norwegian breed graze the pasture as well.

The number of sheep at Buøya was estimated to 2.70 LU. Fourteen percent of the island (5 ha) was pasture and according to Rekdal (7) could carry around 2.5 LU. With a LU density of 2.70, density may be a limiting factor for lambs' growth, since the rest of Buøya is dominated by heath vegetation types and classified as having low nutritional value. Lambs' growth rate was significantly lower on this island compared to Sandvær, but higher than on Sjonøya. When the stocking rate is higher than the estimated capacity of the high nutritional value vegetations types, animals are forced to graze in medium and low nutritional value vegetations types. Species such as purple moor-grass (*Molinia caerulea*) and Viviparous sheep's-fescue (*Festuca vivipara*) were found in the coastal heath vegetation type at Buøya. These species could be important for animals grazing in areas dominated by low nutritional value classes (Haugen, unpublished). Comparing LU and cover of high nutritional vegetation types between Buøya and Sandvær, one could expect a higher difference in lambs' average daily gain. As discussed, parts of the high nutritional areas of Sandvær was not grazed due to the low stocking rate. We suspect that the total area was reduced in forage quality during the summer. On the other hand, the smaller area of high nutritional vegetation types at Buøya could be more intensively grazed and thus maintain a higher quality throughout the grazing period. The investigated islands all had a high degree of plant species diversity. Over a 3-months period, the nutritional value-change would be species-specific and influences by general phenological development as well as the within-year impact of grazing.

The climate along the Norwegian coast is dominated by mild winters and wet summers. The average summer temperatures on the islands are 1–2 degrees lower than that observed at the weather station of Tjøtta (Lind, not published). Steinheim et al. (16) and Nielsen et al. (17) found that local weather affected growth of the lambs over summer, but that the effects were area specific. Nielsen et al. (18) examined the relationship between weather and lambs' growth at Tjøtta farm for 17 years and found that a warm July had a positive effect on lamb growth. Precipitation did not seem to have any direct influence on lambs' growth (18). In the present experiment, 2012 was in general cooler (11.8°C in average during June, July, and August) than the years 2013 (13.4°C) and 2014 (15.4°C) with less precipitation (164, 319, and 171 mm for 2012, 2013, and 2014, respectively). Hatten et al. (8) in a 1999–2001 study in the Vega archipelago, situated about 80 km south of our study area, studied lambs' growth on four islands. The summer of 2001 was warm and dry and affected lambs' growth rate adversely. The animals in that study were collected from the islands late in August when available pasture and fresh water was inadequate and the average daily gain during the last month was negative for some lambs. The islands in our study were larger than the ones used in Hatten et al. (8) and thus likely not as sensitive to the summer weather conditions. We suggest that weather, within the range observed, did not strongly influence lamb growth rates in our study.

Similar challenges linked to phenological development of plants are not found in mountainous areas to the same extent (7). On the contrary, among the benefits of using mountain pastures are the diverse vegetation, the young phenological stages of plants, high in nitrogen, and low in fiber resulting from the snow line retreating upwards. This allows the animals to follow and graze on high quality pastures during the summer.

Lambs' daily gain during summer on mountain pastures varies and depends primarily on factors affecting available forage quality and stocking rate (19). Nielsen et al. (18) found lambs' daily gain both on lowland and mountain pastures to vary between 0.25 and 0.31 kg d^−1^. Animalia (20) report average Norwegian White Sheep lamb daily gain during summer of 0.29 kg d^−1^. This is in the same range as what we found, with an estimated daily gain of 0.32 kg d^−1^. This figure concurs with Hatten et al. (8) who reported lambs' daily gain between 0.25 and 0.33 kg d^−1^ from islands in Vega archipelago.

A dynamic management plan when using island pastures is important. As the islands are flat, phenological development is uniform across the pastures and the stocking rate should ideally be higher in the spring and early summer than later. During the summer, the lambs' need for high-quality forage increase while at the same time the pasture quality declines, decreased digestibility, and crude protein content. However, the pasture quality can to some extent be maintained if the stocking rate is adjusted during the grazing season. To release and collect the animals at the right time are therefore critical for the production output.

## Conclusion

In the present study we evaluated lamb performance on three islands with different grazing value and stocking rates. These lambs had a daily weight gain similar to the average weight gain for the Norwegian White breed on a national level. The homogenous topography and low altitude variation on the islands result in a uniform vegetation development and render the vegetation more sensitive to between and within summer climate variation. Adjustment of stocking rate, date of release, and collection of animals must be fine-tuned. With a dynamic and adaptive management strategy, there are high potential benefits for increasing the use of island pastures.

## Data Availability Statement

The raw data supporting the conclusions of this article will be made available by the authors, without undue reservation.

## Author Contributions

VL was responsible for study design, data collection, analysis, and interpretation, and was the principle author of the manuscript. ØH was responsible for study design, data collection, analysis, and interpretation. F-AH was responsible for vegetation mapping and interpretation. GS was responsible for statistics, analysis, and interpretation. All authors contribute to manuscript revision and have read and approved the manuscript.

## Conflict of Interest

The authors declare that the research was conducted in the absence of any commercial or financial relationships that could be construed as a potential conflict of interest.
